# Neuroprotective effect of Tagara, an Ayurvedic drug against methyl mercury induced oxidative stress using rat brain mitochondrial fractions

**DOI:** 10.1186/s12906-015-0793-2

**Published:** 2015-08-12

**Authors:** Dhanoop Manikoth Ayyathan, Rajasekaran Chandrasekaran, Kalaivani Thiagarajan

**Affiliations:** School of Biosciences and Technology, VIT University, Vellore-632014, Tamilnadu, India

**Keywords:** Methyl mercury, Oxidative stress, Tagara, Mitochondrial viability, Neuroprotection

## Abstract

**Background:**

Methyl mercury (MeHg), an important environmental toxicant is implicated in neurological disorders such as Hunter-Russell syndrome and Autism. Therefore, the present work is in search of new drugs that can alleviate MeHg toxicity. In this connection, Tagara, an ayurvedic drug is used for assessing its neuro protective effect against MeHg toxicity.

**Methods:**

In the present study, we assessed the phytochemical contents of Tagara by colorimetric and HPLC analyses. The neuroprotective effect of Tagara on MeHg induced neurotoxicity was measured in terms of viability by MTT assay and oxidative stress in terms of catalase activity, glutathione and thiobarbituric acid reactive substance levels. Further, the chelating effect of Tagara towards MeHg was performed to identify the molecular mechanism. Statistical analysis was done by statistical package for social sciences (SPSS) version 16.0.

**Results:**

The results demonstrated that Tagara contains significant amounts of phenols and flavonoids. Also, HPLC analysis of Tagara revealed the presence of essential oils such as hydroxyvalerenic and valerenic acids. Our results demonstrated that exposure of rat brain mitochondrial fractions to MeHg resulted in a dose dependent death in MTT assay and IC_50_ value was found to be 10 μM. However, a 250 μg dose of Tagara effectively prevented MeHg induced mitochondrial damage. The oxidative stress caused by MeHg results in elevated levels of reactive oxygen species as evidenced by elevated TBARS (Thiobarbituric acid-reactive substances) levels and diminished catalase enzyme activity and glutathione content. However, Tagara at 250 μg concentration offsets these alterations caused by MeHg. Further, Tagara also diminished GSH oxidation caused by MeHg, confirming its chelating effect, one of the molecular mechanisms that triggers protection against oxidative damage.

**Conclusion:**

Our results revealed that MeHg induced toxicity is predominantly mediated through oxidative stress mechanism and the propensity of Tagara to abolish such reactions. Hence, we propose that Tagara with a source of potential neuroprotectants may be a useful approach to alleviate MeHg associated neurotoxicity.

## Background

Mercury is one of the potential environmental xenobiotic toxicants that is implicated in long-term neurological and developmental disorders in both animals and humans [1, 2]. Among different forms of mercury, MeHg (organic form) is an important neurotoxicant due to its higher entry rate into the CNS compared with inorganic mercurials [3, 4]. Human exposure to methylmercury (MeHg) through consumption of sea foods results in autism spectrum disorders, Alzheimer’s disease, Parkinson’s disease, Huntington’s disease, Minamata disease epilepsy, depression, mood disorders and tremor [4–8].

Current focus of research in the neurotoxicology field is to identify the underlying mechanism behind mercury-induced neurotoxicity and also to find out the antidotes to counteract the toxic effects. Among the three important molecular mechanisms of MeHg-induced neurotoxicity such as oxidative stress, disruption of calcium homeostasis and impairment of glutamate homeostasis, evidences pointed out that oxidative stress, particularly in mitochondria is a central mechanism [6, 8].

Though there are researches related to development of drugs, the treatment for neurological disorders remains wretched due to failure of regeneration of central nervous tissues by modern drugs and their adverse side effects. Because oxidative stress is a common phenomenon in metal toxicity, it is important to understand the interaction between antioxidants and neurotoxicants such as methyl mercury. The benefits associated with intake of antioxidants have been attributed mainly to the antioxidant activity of medicinal plants. Also, some of the Ayurvedic herbs such as *Acorus calamus, Nardostachys jatamansi, Herpestis monniera etc.,* are effective in the treatment of most of the neurological disorders [9].

In this regard, Tagara (*Valeriana wallichii* of family Valerianaceae), an ayurvedic drug that possesses protective effects on several aspects of brain and nervous conditions is chosen for the present study. It is well used in various pharmaceutical preparations for the treatment of migraine [10, 11] , insomnia and anxiety [12]. Tagara is also reported to have antioxidant effect [13]. Although studies have documented the various pharmacological activities of Tagara, very little is known about its interaction with MeHg induced neurotoxicity. Therefore, the intention of the present study is to find out the therapeutic response of Tagara, an ayurvedic drug against methyl mercury induced neurotoxicity. The antioxidants such as phenols and flavonoids present in Tagara were assessed by colorimetric method and the presence of essential oils was confirmed through HPLC. The cell viability was assessed by MTT assay and catalase, glutathione and TBARS levels were also assessed. Also, the chelating effect of Tagara towards MeHg is performed that revealed the molecular mechanism of action of Tagara against MeHg induced oxidative stress.

## Methods

### Animals

Adult Wistar rats (9 months old) were bred in the animal house of Vellore Institute of Technology. All the experimental procedures were carried out in accordance with committee for the purpose of control and supervision of experiments on animal (CPCSEA) guidelines, and all experiments were approved by the institutional animal ethics committee (VIT/IAEC/VII/14). The animals were maintained at 23 °C on a 12 h light/dark cycle with free access to water and food (VIT University, Vellore, India).

### Chemicals

Methylmercury (II) chloride, 5, 5′-dithiobis-(2- nitro benzoic acid) (DTNB), glutathione (GSH)-reduced form and 3-(4, 5-dimethyl-2-thiazolyl)-2, 5-diphenyl-2H-tetrazolium bromide (MTT) were purchased from Sigma. All other chemicals used were of analytical grade.

### Plant sample preparation

Tagara, a whole plant powder in the form of capsules was procured from Himalaya Company, Bangalore, India. Tagara powder of 1 mg/ml concentration was prepared and was determined for total phenolic and flavonoid contents.

### Total phenolic content

The amount of total phenolics present in Tagara was determined using Folin-Ciocalteau (FC) reagent as described using gallic acid as standard [14]. To different concentrations of Tagara, FC and sodium carbonate were added and incubated at room temperature for 20 min. Then, the absorbance was measured at 730 nm. Values are represented as mean ± S.E (*n* = 3).

### Total flavonoid content

The amount of total flavonoids present in Tagara was determined using quercetin as standard [15]. To different concentrations of Tagara, potassium acetate and aluminium nitrate were added. After 40 min of incubation at room temperature, the absorbance was measured at 415 nm. Values are represented as mean ± S.E (*n* = 3).

### Sample preparation for HPLC analysis

For HPLCanalysis, 2 g of fine powder of Tagara was weighed and transferred to a 100 mL volumetric flask. It was then diluted to volume with methanol: water (80:20) and sonicated for 30 min. Then, it was filtered through a 0.45 μm membrane filter [16].

### HPLC instrumentation

HPLC experiments were performed on a YungLin HPLC system equipped with Phenomenex Luna C18, 5 mm (4.6 × 250 mm) column, LC10AT VP pumps, SCL-10AVP system controller, SIL-10 AD VP auto injector, SPD-M10 AVP photodiode array detector and class VP software was used [16].

### Analytical method for HPLC analysis

In this method, the mobile phase used was a mixture of methanol and 0.5 % phosphoric acid (80:20). The following parameters were set such as flow rate of 1.5 ml/min, column temperature of 30 °C and detection wave length at 225 nm. The injection volume used was 20 μl and the total run time was 15 min. The chromatographic system was equilibrated by the mobile phase. [16].

### Sample preparation for neuro protective property

Mitochondrial enriched fractions were prepared essentially as described by Franco et al. [17]. Reaction mixture (R1) of 150 μL was prepared by incubating 2 mg of mitochondrial enriched protein with different concentrations of MeHg (0, 5, 10, 25, 50, 75 and 100 *μ*M) and of Tagara (10, 25, 50, 100, 250 and 500 *μ*g) in a medium containing 5 mM HEPES buffer (pH 7.0), 110 mM mannitol, 34 mM sucrose, and 5 mM KCl for 30 min at 25 °C. After incubations, following parameters were assessed.

### Total protein

Protein concentration was determined by using Lowry method [18].

### MTT assay

Mitochondrial function was assessed by MTT assay [19]. The reaction mixture (R1) of 150 *μ*L was incubated with 150 *μ*L of 1.2 mM MTT for 30 min at 25 °C. The purple formazan crystal pellets were dissolved in DMSO, and the colour obtained was measured at 550 nm. The results were expressed as percentage viability.

### Glutathione content

Glutathione content was measured by the method of Ellman [20]. The reaction mixture (R1) of 150 *μ*L was incubated with 600 *μ*L of 10 % trichloroacetic acid. Then it was centrifuged at 4000 rpm for 10 min at 4 °C. To the supernatant, 300 *μ*L of Ellman’s reagent and 600 *μ*L of 0.1 M phosphate buffer were added. The yellow color developed was read at 412 nm.

### TBARS levels

Lipid peroxidation levels were measured as thiobarbituric acid reactive substances (TBARS) according to the method of Ohkawa [21]. To 150 μL of reaction mixture (R1), 0.375 % 2-thiobarbituric acid, 5 % trichloroacetic acid and 0.25 N HCl were added and was incubated at 95 °C for 60 min. Then the tubes were centrifuged at 1000 rpm for 10 min, and the pink color developed was estimated at 535 nm.

### Catalase assay

Catalase enzyme was measured according to the method described by Sinha [22]. To 150 μL of reaction mixture (R1), 1.35 ml of 0.01 M phosphate buffer (PH 7.0) and 600 *μ*L of 0.2 M hydrogen peroxide were added. After 30 s, 3 ml of dichromate acetic acid mixture (1:3) were added and then heated in a boiling water bath for 10 min. The green color developed was read at 620 nm.

### Chelating effect

The chelating effects of Tagara towards MeHg were analyzed by an indirect method. 10 *μ*M concentration of MeHg was incubated with GSH (100 *μ*M) in the presence or absence of Tagara (250 μg) at 25 °C for 30 min. Then the amount of reduced form of GSH was determined by reaction with 5, 5′-dithiobis-(2-nitrobenzoic acid) that indirectly gives the measure of free mercury [20].

### Statistical analysis

Statistical differences among groups were analyzed by one-way analysis of variance followed by Duncan’s multiple range tests when required. Pearson analysis was used to correlate variables. Differences were considered statistically significant when *P* < 0.05.

## Results

### Total phenol content (TPC)

Phenolics play a vital role in the free-radical scavenging properties of plants. The total phenolic content in Tagara was found to be 185 mg of Gallic acid equivalents per gram of Tagara (Table 1). (Standard curve equation: y = 0.069×, r^2^ = 0.992).

**Table 1:** Total phenolic and total flavanoid contents of Tagara

Name	TPC (mg of GAE/g of extract)	TFC (mg of Quercetin equivalents/g of extract)
Tagara	185 ± 0.07	110 ± 0.4

### Total flavonoid content (TFC)

The total flavonoid content found in Tagara was found to be 110 mg of Quercetin equivalents per gram of Tagara (Table 1). (Standard curve equation: y = 0.139×, r^2^ = 0.998).

### HPLC analysis

**Fig. 1:**
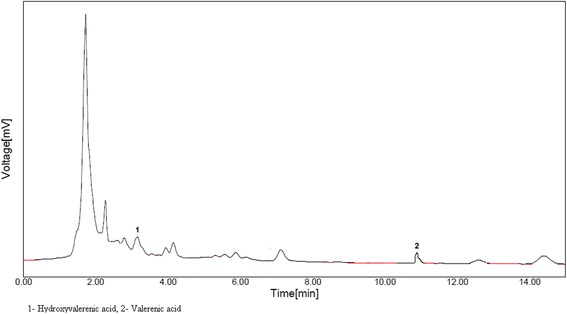
HPLC Chromatogram of Tagara. 1. Hydroxyvalerenic acid, 2. Valerenic acid

The presence of essential oils at 225 nm by HPLC was determined in Tagara and compared with published data [16]. By comparing the retention times of the standards, following compounds (Table 2, Fig. 1) were identified in Tagara *viz*, (hydroxyvalerenic acid and valerenic acid).

**Table 2:** Compounds and Retention times

Peak no.	Compound name	Retention time (Minutes)	Retention time (Minutes) of standards	Reference
1	Hydroxyvalerenic acid	3.56	3.60	Roy*,* 1999
2	Valerenic acid	11.15	11.31

### Assessment of mitochondrial viability

**Fig. 2:**
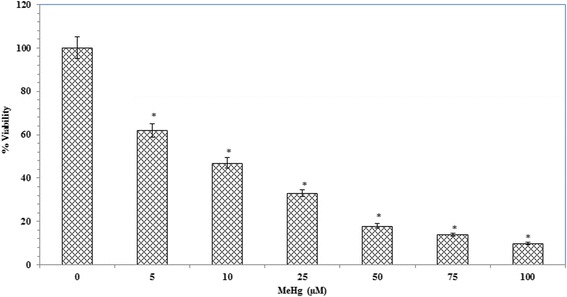
Influence of MeHg on mitochondrial function. Data are expressed as viability percentage. Values are represented as mean ± standard error. *indicates statistically different from control (*P* < 0.05)

**Fig. 3:**
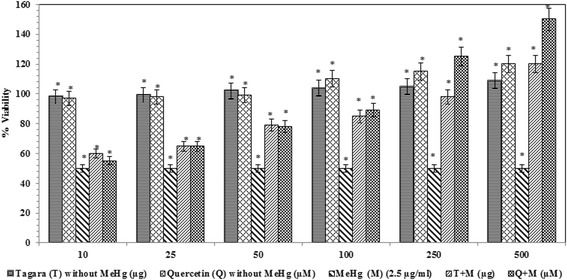
Neuroprotective property of Tagara against MeHg-induced mitochondrial dysfunction. Data are expressed as percentage of viability. Values are represented as mean ± standard error. *indicates statistically different from control (*P* < 0.05)

In MTT assay, our results indicated that MeHg decreased the mitochondrial activity in a dose-dependent manner (Fig. 2) and IC_50_ value was found to be 10 μM. However, Tagara at 250 *μ*g concentrations and positive control quercetin at 250 *μ*M restore MeHg induced mitochondrial dysfunction (Fig. 3).

### Protective effect of Tagara against MeHg induced oxidative stress

**Fig. 4:**
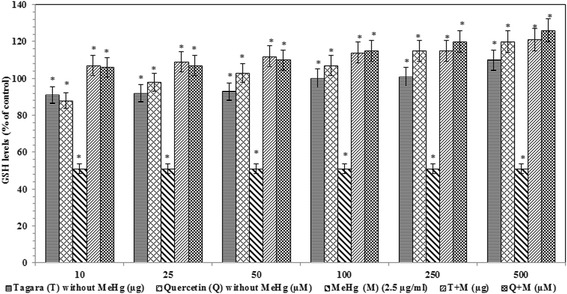
Glutathione contents in treated and untreated groups with Tagara. Data are expressed as percentage of control. Values are represented as mean ± standard error. *indicates statistically different from control (*P* < 0.05)

The glutathione level was found to be reduced significantly to 51 % (*P* < 0.05) when MeHg was used (Fig. 4). However, Tagara at 10 *μ*g concentration and quercetin at *10 μM* concentration have completely prevented this reduction in glutathione levels.

**Fig. 5:**
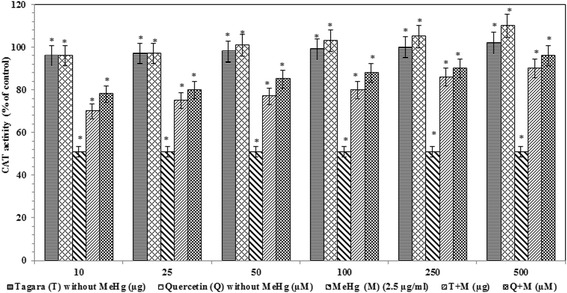
Catalase activity in treated and untreated groups with Tagara. Data are expressed as percentage of control. Values are represented as mean ± standard error. *indicates statistically different from control (*P* < 0.05)

The activity of catalase was significantly reduced in MeHg intoxicated group vs. the control group by −49 % (*P* < 0.05). After treatment with Tagara and positive control quercetin, there is a significant improvement in the catalase activity *viz,* +39 and +45 %, respectively (Fig. 5).

**Fig. 6:**
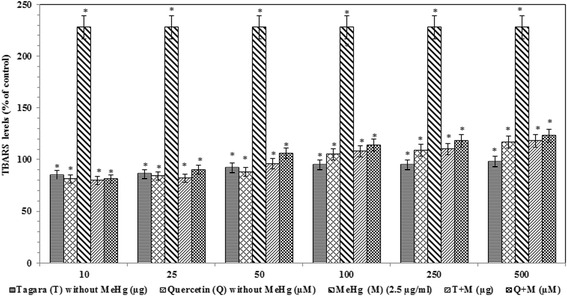
TBARS levels in treated and untreated groups with Tagara. Data are expressed as percentage of control. Values are represented as mean ± standard error. *indicates statistically different from control (*P* < 0.05)

The TBARS levels are significantly increased in the MeHg treated group compared with the control group by +128 %. After administration of Tagara and positive control quercetin, a significant reduction (*P* < 0.05) was noted compared with the control by +8 and +14 %, respectively (Fig. 6).

### Chelating effect

In order to investigate the chelating effect of Tagara against MeHg, the amount of “free” mercurial in the reaction medium was indirectly measured by using reduced glutathione content. Table 3 shows that Tagara at 250 *μ*g concentration was effective in chelating MeHg. The presence of Tagara in the reaction medium changed the oxidative capability of MeHg that converts reduced glutathione to oxidized form.

**Table 3:** Chelating effect of Tagara

	Level of GSH (μmol)	
	Without Tagara	With Tagara at 250 μg
GSH without MeHg	100 ± 1.0	98 ± 2.0
GSH with 10 μM MeHg	46 ± 1.5*	60 ± 1.0*

Student t test showed significant differencce

*indicates, *P* < 0.001, *n* = 3

## Discussion

Many heavy metals including mercury have the ability to produce ROS which initiate chain reactions that provoke oxidation of biomolecules such as lipids, proteins and nucleic acids [23]. Antioxidants defend against metal toxicity by trapping free radicals and or by chelating metal ions [24]. Therefore, the antioxidants such as total phenolics and flavonoids present in Tagara are estimated in the present study. Our study showed that there is a significant amount of phenolics and flavonoids in Tagara, which are thought to be the potential neuro protectants and thus can be used in the treatment of oxidative stress related neurological diseases.

In addition, the essential oils are also reported to have neuro protective properties. The HPLC results of our study also substantiate the presence of essential oils such as hydroxyvalerenic acid and valerenic acid in Tagara. Previous reports also indicated that the roots and rhizomes are highly aromatic and contain valerenic acid that has been shown to inhibit the breakdown of neuro transmitter gamma-aminobutyric acid (GABA) that results in sedation [25].

To study the neuroprotective property of Tagara, isolated rat brain mitochondria a major target for MeHg induced oxidative stress was used as a model [26]. ROS which are produced as a result of normal oxygen metabolism at higher concentration are important mediators of oxidative stress and cell damage [27]. Our results also showed that oxidative stress, induced by MeHg results in significant reduction in mitochondrial function. Previous reports also suggested the same [10, 26].

However, the deleterious effects of ROS are counterbalanced by both antioxidant enzymes such as catalase, superoxide dismutase and non-enzymatic antioxidants such as glutathione, vitamins, flavonoids, phenolics *etc.* [24].

Excessive free radicals play an important role in neural disorders and therefore medicinal plants with rich sources of antioxidants are given considerable importance as they are the important scavengers of free radicals. Many reports suggested that neuroprotective mechanism of bioflavonoids is partly related to their metal chelating and antioxidant properties [28]. Plants also contain important therapeutic metabolites that are involved in brain cell regeneration. Previous study also confirms that the bioactive compound turmerone in turmeric increases neural stem cell growth in the brain and helps in differentiation of neural stem cells to neurons [29].

Therefore in our study, we sought Tagara, an important medicinal herb is responsible for protection against MeHg induced neurotoxicity, using mitochondrial enriched rat brain fractions. Our results showed that MeHg induced oxidative stress results in mitochondrial damage by decreasing the defense mechanisms such as reduced glutathione (GSH) content and catalase enzyme levels. This GSH depletion is due to the fact that MeHg interferes with the uptake of cystine, which is a precursor of GSH synthesis [30, 31] and its interaction with GSH as it has high affinity with SH-groups of GSH [32, 33]. The protective effects of GSH are related to its antioxidant capacity and its ability to conjugate with MeHg for its efflux [34] and therefore its depletion results in cell damage and neurotoxicity. It is interesting to note that there is an increase in GSH content by Tagara (250 *μ*g concentration), supposed to be a promising neuroprotective agent against MeHg-induced neurotoxicity.

Reports also suggested that, catalase an enzyme that detoxifies hydrogen peroxide are reduced in response with MeHg whereas, Tagara efficiently restored catalase activiy. This is in accordance with our previous study with Brahmi [35]. Hence, we could presume that the elevation of GSH and catalase enzyme could be one of the major mechanisms by which Tagara counteracts MeHg toxicity.

It is suggested that oxidative injury, especially lipid peroxidation, via a powerful oxidant (e.g., hydroxyl radicals), may play an important role in cerebellar degeneration during MeHg intoxication. In the present study, our results indicated that MeHg increased the levels of thiobarbituric acid reactive substances (TBARS), a marker of lipid peroxidation. It has been previously reported that the elevation of TBARS is due to an oxidative injury by hydroxyl radicals [36]. Although the levels of TBARS were enhanced in MeHg induced neurotoxicity, Tagara decreased these levels in a significant manner.

In addition to the antioxidant properties of Tagara, the ability to chelate mercurial ions represents an important mechanism for neuroprotection. In fact, our results showed that (Table 3) Tagara is able to chelate metal ions and reinforce its therapeutic potential against MeHg intoxication.

## Conclusion

The data reported here strengthens the antioxidant and protective proficiency of Tagara against MeHg neurotoxicity. Considering the facts that oxidative stress has been implicated in MeHg toxicity and medicinal plants are effective in counteracting the toxic effects of MeHg, the use of Tagara may represent an important therapeutic approach. The present study shows that GSH oxidation is a critical phenomenon involved with MeHg induced mitochondrial dysfunction and that the protective effect of Tagara on such process is directly related to its chelating effect. The phytochemical and HPLC analyses showed the presence of phenols and flavonoids, as well as essential oils. These results indicated that Tagara exerts a significant protection against MeHg induced *in vitro* neurotoxicity. However, these issues need further investigations on the molecular mechanisms associated with mercurial toxicity and the pharmacological studies of Tagara with respect to mercurial poisoning through cell lines and *in vivo* studies.

## Abbreviations

DTNB: 5′-dithiobis-(2- nitrobenzoic acid)
GABA: Gamma-aminobutyric acid
GSH: Glutathione﻿
HPLC: High performance liquid chromatography
MeHg: Methyl mercury
MTT: 3-(4,5-dimethyl-2-thiazolyl)-2,5-diphenyl-2H-tetrazolium bromide
ROS: Reactive oxygen species
TBARS: Thiobarbituric acid reactive substances
TPC: Total phenol content
TFC: Total flavonoid content
